# Skeletal Muscle Metastasis to Vastus Lateralis from a Urothelial Carcinoma: A Case Report and Review of Its Diagnosis and Management

**DOI:** 10.1155/2019/8923780

**Published:** 2019-12-03

**Authors:** A. M. Mainwaring, H. Wells, T. Banks, T. Ellul, P. Bose

**Affiliations:** Department of Urology, Morriston Hospital, Swansea, UK

## Abstract

Bladder cancer is a common genitourinary tract malignancy. Urothelial carcinoma is the most frequent type of bladder cancer and it commonly metastasises to lymph nodes, bone, lung and liver by a haematogenous route. Skeletal metastases are very rare and are usually present in patients with advanced metastatic disease. We present an unusual case of a 71-year-old male with a urothelial carcinoma metastasis to the vastus lateralis muscle 3 months following a cystoprostatectomy for muscle invasive bladder cancer.

## 1. Introduction

Bladder cancer is the ninth most common cancer worldwide, with an estimated 430,000 new cases in 2012 [1]. Urothelial carcinoma (UC) is the commonest type of bladder cancer and greater than 60% of newly diagnosed urothelial carcinomas of the bladder are nonmuscle invasive [2]. However, 50−70% of tumours reoccur and 10−30% progress to muscle invasive disease within 5 years [3]. Bladder cancer metastases to skeletal muscle are very rare and only a small number of cases have been reported in the literature. We present a rare case of a skeletal muscle metastasis to the vastus lateralis muscle from a urothelial carcinoma of the bladder. The authors have obtained the patient's informed written consent for electronic and print publication of the case report.

## 2. Case Report

**Figure 1:**
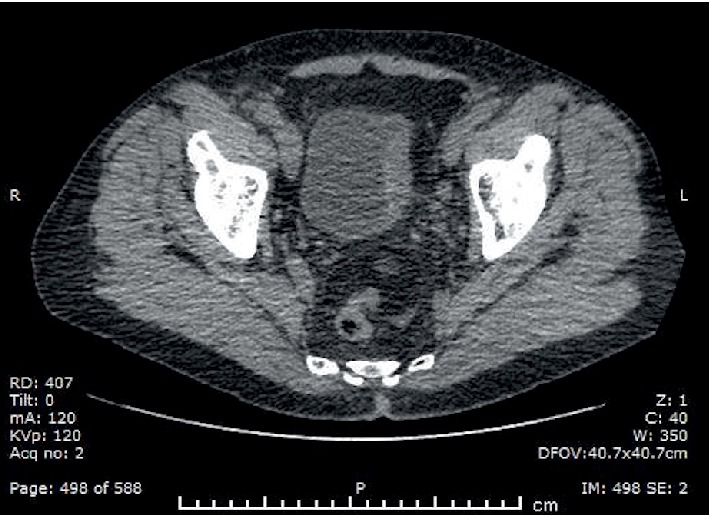
Computed tomography image of a bladder tumour on the left bladder wall.

**Figure 2:**
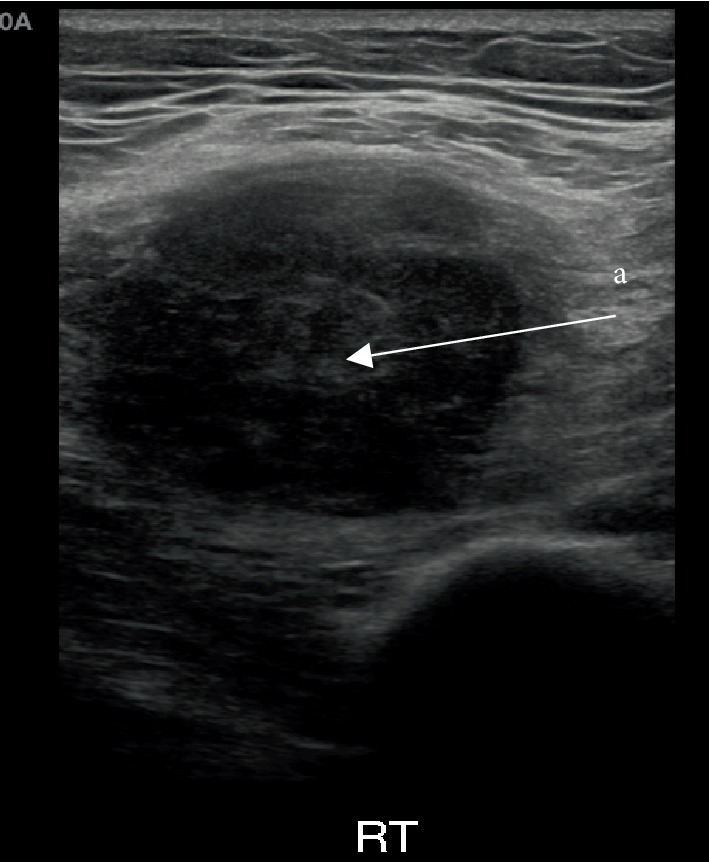
Ultrasound image of a mass in the right vastus lateralis muscle. a: Hypoechoic lesion in right vastus lateralis muscle.

**Figure 3:**
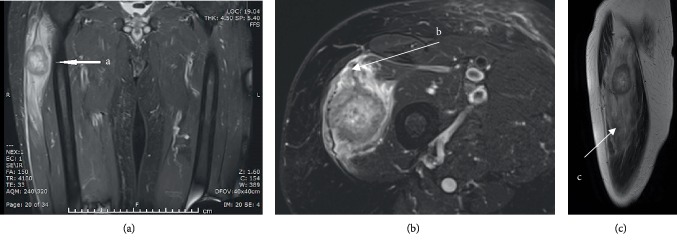
T2-weighted MRI images of the right thigh tumour. (a) Coronal image of the right thigh tumour. (b) Axial image of right thigh tumour and surrounding oedema. (c) Sagittal image of right thigh tumour with enhancing central element.

A 71-year-old male attended haematuria clinic in 2017 with a short history of painless visible haematuria that first occurred on holiday in Vietnam. He has a background of a skin melanoma of the right leg which was completely excised. An ultrasound scan of the bladder and flexible cystoscopy was performed which showed a large bladder tumour on the left bladder wall. A staging computed tomography (CT) scan of the thorax, abdomen, and pelvis was negative for metastases, only the bladder tumour was observed (Figure 1). A transurethral resection of the bladder tumour was performed which showed muscle invasive disease. He subsequently underwent 3 cycles of neoadjuvant chemotherapy (gemcitabine and cisplatin), followed by a radical cystoprostatectomy and ileal conduit urinary diversion. Histological analysis confirmed a high grade muscle invasive urothelial carcinoma of the bladder with perineural invasion (pT3aN0). Three months after his surgery, he attended a follow-up clinic complaining of a painful right hip and a swelling in his right thigh worse on flexion at the hip. There were no skin changes or neurovascular compromise to the limb. Clinical examination revealed a diffuse swelling in the lateral compartment of the thigh. An ultrasound of the right thigh revealed a 43 mm × 31 mm hypoechoic, well circumscribed lesion within the fibres of vastus lateralis (Figure 2). A subsequent contrast enhanced magnetic resonance imaging (MRI) scan showed a 37 × 36 × 48 mm lesion of altered signal intensity with limited central enhancement, suspicious of a skeletal muscle metastasis (Figure 3). He also developed bone metastases at the L3 vertebra and right pubic bone. An ultrasound guided biopsy of the right thigh lesion was identified as a poorly differentiated carcinoma with positivity for pankeratin and cystokeratin 7 consistent with a urothelial carcinoma and confirming the diagnosis of skeletal muscle metastasis. He was referred to oncology and underwent 4 cycles of anti-PD-L1 monoclonal antibody immunotherapy (IV atezolizumab 1200 mg one dose per cycle) as part of a clinical trial. Despite better pain control and reduced right thigh swelling, follow up imaging suggested a slight increase in the size of the metastatic deposit in the thigh. He subsequently received a course of palliative radiotherapy to the lumbar spine, pelvis, and right thigh (20 Gy in 5 fractions at each site) followed by six 14 day cycles of IV paclitaxel chemotherapy (175 mg/m^2^/day). The patient demonstrated partial remission as the skeletal muscle metastasis reduced in size to 22 × 34 × 52 mm (including an area of tumour necrosis) assessed by a repeat MRI scan in March 2018. However, the scan identified a new 53 × 40 × 18 mm subcutaneous UC metastasis over his left scapula which was fully excised, and the site was irradiated (20 Gy in 5 fractions). The patient continues to make good progress and he is mobile and self-caring. His disease to date has remained stable on follow up imaging and he is currently under CT surveillance.

## 3. Discussion

The commonest type of bladder cancer is UC accounting for over 90% of bladder tumours, 5% are squamous cell carcinomas and fewer than 2% are adenocarcinomas [4]. The commonest sites of metastases from bladder cancer are lymph nodes, bone, lung, liver and peritoneum [5]. Other unusual metastatic UC sites include the brain, skin and pericardial effusion have been reported [6–8]. Skeletal muscle metastases (SMM) are rare, despite muscle constituting up to 50% of total body mass and having a substantial blood supply [9]. Multiple factors have been hypothesised that limit the risk of metastatic spread to skeletal muscle. These protective factors include muscle pH, muscle contractility, alterations in oxygenation, and lactic acid accumulation that may reduce risk of metastatic tumour development [10, 11]. Magee and Rosenthal [12] reported that skeletal muscle trauma may increase the propensity for developing metastases within the affected muscle due to a change in muscle physiology that disrupts the previously described protective mechanisms. Other primary malignancies have been reported to metastasise to muscle such as the lung, kidney, stomach, pancreatic, colon and ovarian [13, 14]. The true incidence of muscle metastases may be underestimated. Autopsy studies have reported a higher incidence of muscle metastases ranging from 0.8% to 16% [15]. The number of patients who harbour muscle metastases may be higher in advanced stages of disease but only a few survive their disease long enough to present with clinical signs and symptoms from the affected site [13].

Metastases to skeletal muscle from UCs are extremely rare and only a few cases have been reported in the English literature [9, 16–21]. Nagao et al. [14] reported a case of a gluteus maximus metastasis from an adenocarcinoma of the bladder. A large proportion (50%) of muscle metastases from a carcinoma are found in the lower extremity [13]. Nabi et al. [22] reported UC metastases in the adductor, psoas, and rectus abdominis muscles. To date, our case is the first report of a biopsy proven UC metastasis to the vastus lateralis muscle. In all of the reported cases including our own, the presenting features were of a localised, painful swelling within the affected muscle.

It is difficult to distinguish between a metastatic carcinoma to the muscle and primary soft tissue sarcoma based on clinical examination or imaging studies [13]. Imaging can be used to help identify SMM and guide tumour biopsy. The CT features of SMM have been described as low density, ring enhancing lesions by Nabi et al. [22]. Surov et al. [23] described 5 different types of SMM based on CT appearances: focal intramuscular masses with homogenous contrast enhancement (type I), abscess-like intramuscular lesions (type II), diffuse metastatic muscle infiltration (type III), multifocal intramuscular calcification (type IV), and intramuscular bleeding (type V). SMM may present similarly to an abscess, therefore clinical corroboration and a high index of suspicion is required to achieve an accurate diagnosis. MRI is superior to CT for imaging soft-tissue tumours [24]. Heterogenous iso-signal intensity in T1-weighted sequences and heterogenous hyperintense signal in T2-weighted sequences with or without adjacent bone invasion with localised swelling and pain have been identified as features of SMM on MRI imaging [25]. Kashyap et al. [18] reported that F-18 FDG PET/CT scans could be useful to identify distant bladder cancer metastases. They observed intense FDG uptake in ring-enhancing lesions in multiple lower limb muscle groups suggestive of skeletal metastases from a primary bladder malignancy. However FDG is not specific for malignant cells and it also shows uptake in inflammatory/infectious sites, hence it is currently not recommended for routine imaging of soft tissue tumours [26]. Image guided biopsy of a suspected SMM is best to establish a diagnosis and hence, provide the most suitable treatment. Currently, there is no absolute immunohistochemistry marker or panel to confirm urothelial differentiation [27]. The uptake of immunohistochemical markers such as GATA3, S100P, CK7, CK20, HMCK, and p63 are suggestive of urothelial lineage of variant morphologies, whilst positivity for GATA3, CK20, p63, and either high molecular weight cytokeratin (HMCK) or cytokeratin (CK)5/6 can be useful in the differential diagnosis of urothelial carcinoma [27, 28].

Treatment of SMM is dependent on tumour location and the performance status of the patient. Chemotherapy, radiotherapy, and metastectomy are treatment options, usually performed for palliation of symptoms rather than with curative intent due to the likelihood of advanced malignant disease already present in the patient. Pretorius et al. [29] reported that 87% patients diagnosed with SMM had evidence of widespread metastatic disease. Patients presenting with a SMM have an average survival of less than 9 months and no more than 3 years after diagnosis, although there are reports of patients surviving up to 5 years after diagnosis [30]. Nabi et al. [22] reported in their series of UC SMMs patient survival was an average of 8 months (range 6−12 months), all 5 patients were treated with palliative chemotherapy and 2 patients also received palliative radiotherapy. Recent advances in immunotherapy have provided an additional treatment option for metastatic bladder cancer and since May 2016, 5 agents that target the programmed cell death 1 (PD-1) pathways have been approved for use following disease progression on platinum-based chemotherapy [31]. The SAUL trial, a multinational single-arm safety study of atezolizumab therapy for locally advanced or metastatic urothelial or nonurothelial carcinoma of the urinary tract for patients who had progressed during or after one to three therapies, has recently published its primary results. It showed that atezolizumab is tolerable and an effective treatment for urinary tract carcinoma, including patients with autoimmune disease and renal impairment [32]. These findings may provide a viable treatment option for this group of patients who may also have multiple co-morbidities, however the final results of the trial are awaited. Our patient received anti-PD-L1 monoclonal antibody immunotherapy, palliative radiotherapy and 6 cycles of chemotherapy. He continues to lead an active life and currently his disease burden is stable.

## 4. Conclusion

A skeletal muscle metastasis should be considered in patients with a history of urothelial malignancy who present with new muscle pain with or without swelling. MRI imaging together with tissue biopsy and a high index of clinical suspicion are the most useful methods of ascertaining a correct diagnosis.
